# Twelve tips for online synchronous small group learning in medical education

**DOI:** 10.15694/mep.2021.000076.1

**Published:** 2021-03-25

**Authors:** Sharon Sneddon, Genevieve Stapleton, Camille Huser

**Affiliations:** 1 University of Glasgow

**Keywords:** Small group learning, Online distance learning, Synchronous, blended learning, COVID-19

## Abstract

This article was migrated. The article was marked as recommended.

Undergraduate medical education relies on a variety of small group learning formats to deliver the curriculum, support collaborative learning, encourage critical thinking, as well as the development of a number of professional, clinical and generic attributes. However, the SARS-CoV-2 (COVID-19) pandemic of 2020 reminded us that unanticipated circumstances may necessitate a rapid and abrupt switch to delivering medical education through alternative means, while still upholding teaching standards and meeting learning and graduate outcomes. For many medical schools, the pandemic resulted in small group teaching being moved to an online format. The experience of students and facilitators moving small group learning tutorials to online synchronous delivery forms the basis for a set of recommendations when considering the delivery of small group teaching remotely.

## Introduction

The COVID-19 pandemic of 2020 necessitated a swift and abrupt adaptation of medical education and its associated pedagogies. This article will focus on the pedagogy of transitioning face-to-face small group learning (SGL) to synchronous online delivery, whilst maintaining the advantages of this important mode of learning for undergraduate medical students.

SGL is a fundamental inclusion in all medical schools, and helps towards the requirement from regulatory bodies, such as the General Medical Council (GMC), to support independent and self-directed learning, ( General Medical Council, 2015). The benefits of SGL are recognised in all fields of education and training and form an essential component of undergraduate medical education ( Jones, 2007; Brandl *et al.,* 2017).

SGL promotes student engagement, critical thinking, collaborative and independent learning, while exploring and challenging attitudes and beliefs ( Edmunds and Brown, 2010). Students also gain a sense of belonging and community, through increased contact time with staff and learning through social interaction ( Mills and Alexander, 2013). Group members develop interpersonal and social skills, and learn to communicate effectively, which are essential skills for the healthcare setting ( Burke *et al.,* 2020). Furthermore, most SGL formats support the development of metacognitive processes and self-regulated learning by formalising reflective practice ( De Grave, Boshuizen and Schmidt, 1996). SGL provides an immediate opportunity for feedback both from peers and tutors, as well as a forum for students to evaluate their learning.

The most common SGL approaches used in undergraduate medical education are: problem-based learning (PBL) ( Barrows and Tamblyn, 1980), case-based learning (CBL) ( Thistlethwaite *et al.,* 2012) and team-based learning (TBL) ( Michaelsen *et al.,* 2009). All three approaches are founded in active learning ( Prince, 2004), but differ in the amount of preparation before class, input and guidance from the tutor and self-direction by students ( Hopper, 2018).

The success of SGL depends on many factors, from the seating arrangement to the skills employed by the tutor to encourage confidence and participation ( Mir, Jeelani and Alshahrani, 2019). A well-functioning group is critical to a successful SGL experience and the tutor has a pivotal role to ensure an environment is created that supports collaborative learning from all group members ( Hendry, Ryan and Harris, 2003).

Medical education has embraced a more inclusive ethos where student diversity is welcomed and supported. Online synchronous teaching delivery can support students by reducing the need to attend all learning opportunities physically. While it is generally accepted that medical education could never be fully delivered online ( Harden and Hart, 2002), the advantages of blended learning, particularly for the delivery of information, can be beneficial ( Graham, Woodfield and Harrison, 2013). However effective learning may suffer if blended learning approaches do not adhere to pedagogical best practice ( Cook, 2007). How can the advantages of SGL be maintained when it is delivered online?

The COVID-19 pandemic saw our tutors and students adapt enthusiastically and successfully to the delivery of online synchronous problem-based learning tutorials. Their subsequent feedback, together with the experiences of the authors in small group delivery (CBL, TBL and PBL), both face-to-face and remotely, form the basis for the following tips to support successful delivery of online synchronous SGL.

## Tip 1 - Adhere to the principles of Small Group Learning pedagogies

With a move to online small group learning, the principles of effective learning design established in face-to-face sessions must be maintained. The processes underlying SGL such as PBL, TBL and CBL are evidence-based, so it is important that students and tutors understand that missing steps will hinder student learning. Moust, Berkel and Schmidt, (2005) identified that learning and the acquisition of knowledge is impacted when students skip steps of the SGL process. In our experience of online synchronous PBL where students are encouraged to explain concepts in their own words and not simply read their notes, students expressed their frustration that their peers were simply regurgitating their notes and not following the agreed process. To mitigate this, tutors were encouraged to ask the group to reflect on the process and use peer feedback to encourage best practice.

## Tip 2 - Re-write the ground rules

The forming of any small group usually begins with the establishment of ‘ground rules’ - what is and is not expected by all group members ( Azer, 2004). Rules regarding attendance, contribution, and respect for all members are commonly agreed upon for most SGL ( Kitchen, 2012). Online groups need to establish the same ground rules, however they become more difficult to ensure equal commitment from the whole group. Our tutors expressed concern that students appeared to be ‘multitasking’ with more than one device, ‘googling’ their contributions, which was affecting contributions and engagement with the process.

As a new group forms (or as part of student training, see Tip 11), the importance of ground rules needs to be made explicit and provide the evidence to students why each rule is important. For example, in PBL, an important part of the process is to activate prior knowledge on a subject as a basis for the construction of new knowledge and learning objectives, which for many students can cause some anxiety ( Maudsley, Williams and Taylor, 2008). Pre-preparation for this step can significantly impact the learning process by disengaging other group members who feel at a disadvantage or less knowledgeable. Explaining the importance of the learning step and an agreement not to prepare as part of the ground rules will help the process.

New ground rules for online SGL might include:

1. Turn all other devices off
2. Keep your camera on whenever possible
3. Adhere to the process

## Tip 3 - Adapt group member roles

Collaboration and student interaction form an essential part of many SGL processes ( Webb, 1982), with both tutors and students having a role to play. The group member’s role is to contribute to the discussion, however online discussions are difficult with time delays, which often lead to talking over each other. This is particularly troublesome when internet connections are poor. Discussion tended to flow less freely, however the use of a ‘hand-raising’ signal to indicate readiness to contribute provided a more seamless change of contributor.

In PBL, there are the additional roles of chair and scribe ( Azer, 2004) which need to adapt to the online format. The role of scribe was noted to be particularly difficult to adapt. The scribe typically records the session on a whiteboard. For the scribe to remain an active participant in discussions, most of our groups modified scribing to making a list of bullet points using an online chat function. This helped the group keep track of issues to discuss, recording important points, while allowing the scribe to remain engaged in the whole process and not be reduced to recording minutes.

As a consequence of modified group member roles, the role of the tutor must adapt to support student participation. While the course coordinator should provide additional training (see Tip 11), the tutor needs to remind and provide guidance on these roles. And finally, the tutor should lead the students to reflect on the process to support further online SGL adaptation and optimisation.

## Tip 4 - Establish and maintain group dynamics

Establishing a well-functioning group is dependent on the formation of new peer-relationships and SGL has the added benefit of providing the opportunity to make friends, something students find challenging in online formats ( Erickson *et al.,* 2020). Building in social time into the SGL session helps to form connections that are more difficult to develop organically online. It is a valued opportunity by tutors and students to connect with each other. For a well-functioning group, make sure all members are visible on screen at the same time, and take the time to explain the importance of the for the group dynamic. Some students turn their camera off and use audio only. In some cases, this was due to a poor internet connection, but for less confident students, it may provide a hiding space from the group. Our students expressed unease when faces could not be seen on the screen and preferred the sense of a ‘whole group presence’ by having videos on. Allocating time at the end of any SGL session, for example the reflection step in PBL, provides the opportunity to address any issues that have arisen that affected the group dynamic.

## Tip 5 - Keep a caring eye on your students

A skilled tutor will regularly monitor student behaviour, body language and facial expressions, all of which helps the tutor to maintain good group dynamics but can also identify students requiring further academic or pastoral support. Students new to university, particularly those starting university during the pandemic, will probably have different expectations of their university experience. There will have been little or no face to face freshers’ events or social interaction which will be disappointing for some, potentially affecting mood and engagement. Tutors should continually monitor engagement levels and any signs of disengagement, or concerns about students and give space for students to share how they are feeling as online learning can be a lonely and isolating experience ( Kaufmann and Vallade, 2020). After each session, invite a different student to stay online for a ‘catch up’, to remind them that support, advice and a friendly face is there whenever they need it.

## Tip 6 - Culture motivation to learn

Our students told us that online synchronous PBL was a strong source of motivation for them to engage in learning activities. Paquette, (2016) showed that students disengage from online learning due to feelings of isolation, frustration and a lack of faculty contact, emphasising the importance of cultivating a community to maximise student satisfaction. Motivation to learn is an important component of self-regulated learning ( Zimmerman, 2008), and is positively associated with student performance ( Richardson, Abraham and Bond, 2012). It is important that students take ownership of their learning, and this can make the learning process enjoyable ( Stipek, 1996). The use of clinical scenarios in SGL to support relevance and application, as well as the socio-constructive basis of knowledge rather than rote learning, tend to make SGL an enjoyable way to learn, increasing learner motivation ( Davis, 1999). Burgess *et al.,* (2019), describe the motivation generated through a community of practice in TBL and conversely how a lack of community can prevent online learning ( Song *et al.,* 2004).

## Tip 7 - Use “breakout rooms” to encourage collaboration and interaction

If software choice allows, an advantage of online synchronous SGL is the possibility to use one tutor for more than one group, relieving staffing pressures to deliver SGL, while allowing students to develop collaborative learning and group work skills independently of the tutor ( Chandler, 2016). This format was particularly useful during the pivot to online delivery during the pandemic as not all tutors were available to change to the online format at short notice. Our experience delivering synchronous online vocational studies tutorials involved small groups of students working through a structured series of active learning tasks with a ‘roving tutor’ between groups. Group expectations are particularly important when one tutor runs multiple synchronous small group sessions using breakout rooms. In this case, the tutor made the expectations of these students clear at the start to work through the activities collaboratively. The tutor could then enter a breakout room, ensure the process was proceeding, and facilitate further discussion. Saltz and Heckman, (2020) took a similar structured approach when using breakout rooms to support the process and saw improved productivity, motivation and student connectedness.

## Tip 8 - Rethink the use of whiteboards

The use of a white board, either to further communicate or explain ideas is frequently used in SGL pedagogies and is an integral part of PBL where the role of scribe is allocated ( Burke *et al.,* 2020). The use of a whiteboard and the role of scribe was perhaps the most challenging aspects to transfer to the online format. Many of our SGL groups reported not bothering to use the whiteboard but both tutors and students recognised the importance of being able to scribe the session, and in particular, use it to draw structures or concepts.

The whiteboard functionality is limited due to the slow speed that text and drawings appear on screen which puts pressure on the time of the group session. In addition, devices used by many students did not allow the ability to write and draw as accurately as they would on a real whiteboard. To overcome this limitation, scribing of main discussion points was undertaken directly in the chat function, described by Hastie, (2008), or by the scribe in a separate text or presentation document. Screensharing was also used to share diagrams and resources which were then discussed by the group. Another option to record discussion topics is the use of mind-mapping software which can then be shared using screensharing functionality.

## Tip 9 - Make online SGL easily accessible

Online SGL should be easily accessible both in terms of technical access by staff and students, and ease of use by diverse abilities and backgrounds. During our pivoting experience, the setup of meeting links was the responsibility of one coordinator, rather than asking tutors or students to set up their own groups. The latter led to slight differences in set up parameters and SGL experiences. For example, students in some groups were able to enter the virtual meeting room early, to socially interact before the arrival of the tutor, while others were permitted to join meetings once the tutor had logged in. Similarly, differences in meeting set up could allow all participants to share screens for some groups, while this was restricted to the tutor for other groups.

Our experience showed that it is also important to use one link for each group which remains the same for all small group meetings, alleviating confusion and preventing students or tutors waiting in the wrong virtual meeting room.

Using software with synchronous closed captioning or recording and providing automatic transcripts can further support accessibility for students and tutors with a range of abilities and backgrounds ( Nordmann *et al.,* 2020). We also offered a range of timeslots for students who may be in different time zones or have caring or work commitments.

## Tip 10 - Choose your software wisely

When deciding which online meeting software to use, the following should be considered:

• Flexibility of device: participants should be able to access the meeting from any web-enabled device (PC, tablet or phone).
• Software should adapt to bandwidth: participants should get the best experience possible with the bandwidth that they have. Adaptive software will reduce the quality of video or even stop the video altogether for example rather than disconnect a participant with fluctuating internet connection.
• Security: ensure that meetings can be kept secure from intrusion by ‘videoconference-bombers’ ( Hodge, 2020). This can be done using passwords to access the meeting, using waiting rooms or locking meeting rooms. However, in our experience, the use of waiting rooms or locking a meeting room once all participants had arrived led to students needing to wait to re-join a meeting after being disconnected.
• Interaction and sharing are vital in SGL, and software should allow students to use whiteboards, split into smaller meeting rooms, share screens and work together synchronously. Breakout rooms have been successfully used in TBL ( Franklin *et al.,* 2016) where larger student numbers are often involved.

## Tip 11 - Train staff and students to adapt to online synchronous SGL

The use of any SGL approach requires training for both faculty and students ( Farmer, 2004), which will need to be further adapted for online synchronous SGL. For tutors, one short virtual training session was sufficient for staff already experienced in face-to-face SGL. Training focussed on the adaptations to SGL mentioned (Tips 1 to 8), ensuring that the differences in online SGL group facilitation were emphasised. Staff training ensured that tutors were able to use the technology to its full potential, allowing as much interactivity as possible without wasting time during tutorials.

Similarly, we produced clear student instructions to ensure they understood the capabilities of the selected software and were able to make maximal use of functionalities. In our experience, student training with an open, co-creation ethos also led to innovative approaches, as students could be consulted on the features which they found most useful.

## Tip 12 - Take an adaptable and flexible approach to online SGL

As with all teaching methods, it is prudent to plan for the unexpected. From our experience, online SGL contingency planning should include spare meeting links to use in case one fails to work and ‘on-call’ tutors to cover groups in case of issues with internet connection for the assigned tutor. The designated coordinator will be responsible for checking the smooth functioning of all the groups. All students and tutors should have the coordinator’s contact details to address and rectify issues quickly.

In the long term, a flexible and adaptable approach is essential. As online meeting innovation rapidly evolves, this flexibility, together with the increased digital experience of both faculty and students will ensure continuous quality improvements. A strong staff-student partnership to utilise the strength of the student digital expertise proved invaluable and will be essential in the future development of online synchronous SGL.

## Conclusions

It seems inevitable that medical education will move towards some form of blended learning as a result of the COVID-19 pandemic. Combining face-to-face with synchronous and asynchronous web-based delivery may well see the final nail in the coffin for the traditional lecture theatre however SGL will remain a necessity for medical education. While physically meeting in a shared space remained the preferred option for small group learning by our tutors and students, the validity and value of online SGL as an equal alternative to face-to-face SGL should be emphasised. With adaptations and a flexible approach (Tips 1-12), online SGL deserves consideration as part of a deliberate blended learning approach in medical education, and not only when necessity (such as a pandemic) requires it.

## Take Home Messages

• Keep the current pedagogy of SGL in mind when adapting for an online format
• Build in systems to create a safe and welcoming online learning environment and maintain student motivation to learn
• Ensure technology remains simple to use on a wide variety of devices
• Train staff and students to ensure an optimised and consistent online SGL experience

## Notes On Contributors

Sharon Sneddon, PhD, is a lecturer in the Undergraduate Medical School. She is an experienced facilitator of problem- and team-based learning, and co-ordinates case-based learning at the University of Glasgow. ORCiD: https://orcid.org/0000-0001-9767-4180

Genevieve Stapleton, PhD, is Year 1 deputy director for the undergraduate MBChB degree, an experienced PBL facilitator and lead for PBL facilitator training at the University of Glasgow. ORCiD: https://orcid.org/0000-0002-7493-5277

Camille Huser, PhD, is a lecturer for the undergraduate MBChB degree and deputy director of the online Health Professions Education Master Programme at the University of Glasgow. ORCiD: https://orcid.org/0000-0002-3785-7556
