# Supplementary material for: Twelve tips for online synchronous small group learning in medical education
Source: MedEdPublish (2016). 2021 Mar 25;10:76. [Version 1] doi: 10.15694/mep.2021.000076.1 (PMC10939637; doi:10.15694/mep.2021.000076.1)
Supplement: Peer review history

# Reviewer report: Reviewer response for version 1

**DOI:** 10.21956/mep.18953.r27124

**Published:** 2021-04-06

**Authors:** Megan Anakin

**Affiliations:** 1 University of Otago

This review has been migrated. The reviewer awarded 3 stars out of 5 Your article caught my eye because one of my roles is as a faculty developer who works with clinical educators that facilitate synchronous small group learning in online environments with undergraduate students in health professions programmes at my university.Anne Tierney makes many helpful suggestions for you to enhance this practical tips article. Like her review comments, my review is focused on ways to improve this article.Since readers may decide if they will read articles by skimming the abstract, please consider summarising the characteristics of the 12 tips by briefly describing the “set of recommendations” in last sentence of the abstract because the other sentences in the abstract provide background information only. The take home messages contain a good summaries of the characteristics that could be used in the abstract.I agree that anchoring your tips in a framework, such as the one Anne suggests, will help readers appreciate how your experiences and examples can be translated to their teaching contexts. For Tip 1, please describe and cite the principles of effective learning design that have been established for in-person sessions that must be maintained and specify how they are the same and different for PBL, TBL, and CBL so the reader can appreciate the modifications you are suggesting for conducting synchronous small group learning in an online environment. These descriptions will help the reader better understand the comparative statements made in Tip 2 about the rules becoming “more difficult to ensure equal commitment from the whole group” and how to establish ground rules of the online environment. For Tip 6, please provide an example to explain how you cultivated motivation to learn with your students in the online synchronous PBL setting mentioned in the first sentence of this tip. In Tip 12, please support absolute statements in the first sentence of the first and second paragraphs with references and please explain how the unexpected can be anticipated and why a flexible and adaptable approach is essential. Please also provide examples or references to more fully describe the ideas presented in the second and third sentences of the second paragraph. To strengthen the conclusion, please consider revising the first sentence to relate directly on the focus of the article: to present 12 Tips that readers can use to enhance their facilitation of synchronous small group learning in an online environment. Please consider omitting the sentence beginning: “While physically meeting …” because the validity and value of online SGL is not the focus of this article.Please consider using simple descriptive language instead of the phrases “pivoting experience” in Tip 9 and “the final nail in the coffin” in the conclusion because an international reader may not be familiar with these colloquialisms.I would be very happy to read and review a revised version of this article.

Reviewer Expertise:

NA

No decision status is available

# Reviewer report: Reviewer response for version 1

**DOI:** 10.21956/mep.18953.r27125

**Published:** 2021-03-25

**Authors:** Anne Tierney

This review has been migrated. The reviewer awarded 4 stars out of 5 First of all thank you for asking me to do this review. I enjoyed reading it very much and welcome its contribution to dealing with online and blended learning due to the pandemic.I have a few suggestions which I think will strengthen the paper. These suggestions come after reading the paper and the thoughts that went through my head as I was reading it, so relate to the flow of the paper, and the clarity, for a first time reader.The first suggestion I have is that you consider using Garrison and Anderson’s Community of Inquiry (CoI) as a framework for the tips. I am suggesting this as it addresses several of the issues that you have picked up on, and frames them for the reader so that right from the beginning, they can see that CoI describes issues that arise in online learning, offers ways to address them, and your tips give concrete examples in SGL. Using CoI will also allow you to strengthen the conclusions by revisiting it and tying your tips and CoI together. Have a read at the papers I’ve linked to and see what you think. I’ve added references to CoI at the end of the review.As I read towards the end of the tips, it struck me that Tips 5, 10 and 11 would benefit from being moved to the beginning of the list. If I think about this as me approaching online SGL as a tutor or a teacher, what would I want to know first? Even before I go into the online space, I’d want to know how do I do SGL online, how do I support my students and how easy is my software to use. Once I was more comfortable about those preparatory things, then I’d want to know what was happening in my session, which is the focus of the other tips. In Tip 3, I noticed that the scribe and chair roles are mentioned, but nothing is said about any of the challenges of being a chair online. It might be an idea to complete the role of the chair by commenting that there weren’t any perceived issues of chairing online. Also, to note, if you move Tip 11 to the beginning, it has already been addressed by the time you get to Tip 3, so there’s no need to refer to it in brackets.In Tip 4 there is a typo – “the importance of the _____ for the group dynamic”.The Tips in general – check that when you have described an issue that you have also proposed a solution. The one that stood out here was Tip 6 – culture motivation, but no suggestions how to do it.I think you could make a bigger deal of your conclusions, as there is actually a lot to digest in the Tips, and the impact and complexity of dealing with SGL online is not given the emphasis it deserves. For any of us moving to online learning in the pandemic, this was really hard, and the work that everyone did deserves to be acknowledged. Going back to my suggestion of using CoI to frame the tips, if you refer back to the CoI at the end, and tie in your tips with the framework, I think this would strengthen the conclusions and the paper over all. I hope you find these suggestions helpful. Thank you again for inviting me to contribute to the review. This has been a tough time for everyone and the tips are a practical contribution, not only in medical education, but for anyone moving towards an online/blended approach.Garrison, D. R. and Anderson, T.. (2003) E-Learning in the 21st Century: A Framework for Research and Practice. London: Routledge/Falmer, 2003.Garrison, D. R., T. Anderson and W. Archer (2000) Critical Inquiry in a Text-Based Environment: Computer Conferencing in Higher Education. The Internet and Higher Education 2(2-3): 87–105, 2000.Andhttps://www.researchgate.net/profile/D-Garrison/publication/248540829_Researching_the_community_of_inquiry_framework_Review_issues_and_future_directions/links/59eb8ae0a6fdccef8b0c59cf/Researching-the-community-of-inquiry-framework-Review-issues-and-future-directions.pdfhttps://d1wqtxts1xzle7.cloudfront.net/48372053/j.iheduc.2009.10.00320160827-32045-slnfo1.pdf?1472361188=&response-content-disposition=inline%3B+filename%3DThe_first_decade_of_the_community_of_inq.pdf&Expires=1616689308&Signature=RGqh1FfmhdvJt51KdAMxjS5GJfq3HCy8WzhtblZ~SJhTJmvwTTN~3klc10JETK0xnxuIiPfUtNDDQHeZCazCn0fWQ0Mf3mkZEtH6avB04pNu0STcF2-FY1qr8NZJn7Qk7V-pGicsbqSno3iIrPgPRPuSbxccFmwV1y1vZfXVi2hy0G1pLNShoTxwUts4P6f6JENioFXQ91GxUZTwNW--0WLzIp~vQD8y~Dxg9XqdhGr6aCN7pGxx-yoKasxS3Mix-AK2vaHMcFGXVHPCvDh0CIEiHSVmYqRYuVNoDgclp1t8Bc-iVgj2KfNs77CeFpQ~7yaFsKFxtvFdGwQ1zbDR2g__&Key-Pair-Id=APKAJLOHF5GGSLRBV4ZA

Reviewer Expertise:

NA

No decision status is available
